# Chitosan/PEO Nanofibers as a Delivery Platform for Sustained Release of *Centella asiatica* Extract

**DOI:** 10.3390/ijms262412134

**Published:** 2025-12-17

**Authors:** Katarzyna Witkowska, Magdalena Paczkowska-Walendowska, Matylda Nagalska, Andrzej Miklaszewski, Tomasz M. Karpiński, Tomasz Plech, Francisco J. Otero Espinar, Judyta Cielecka-Piontek

**Affiliations:** 1 Department of Pharmacognosy and Biomaterials, Poznan University of Medical Sciences, Rokietnicka 3, 60-806 Poznan, Poland; witk.katarzyna@gmail.com (K.W.); 86488@student.ump.edu.pl (M.N.); jpiontek@ump.edu.pl (J.C.-P.); 2 Faculty of Materials Engineering and Technical Physics, Institute of Materials Science and Engineering, Poznan University of Technology, 60-965 Poznan, Poland; andrzej.miklaszewski@put.poznan.pl; 3 Department of Medical Microbiology, Medical Faculty, Poznan University of Medical Sciences, Rokietnicka 10, 60-806 Poznan, Poland; tkarpin@ump.edu.pl; 4 Department of Pharmacology, Medical University of Lublin, Radziwillowska 11, 20-080 Lublin, Poland; tomasz.plech@umlub.pl; 5 Paraquasil Group, Institute of Materials iMATUS and Health Research Institute of Santiago de Compostela (IDIS), University of Santiago de Compostela, 15706 A Coruña, Spain; francisco.otero@usc.es

**Keywords:** *Centella asiatica*, nanofibers, chitosan, electrospinning, wound healing

## Abstract

The search for multifunctional wound dressings that combine structural integrity with biological activity remains an important challenge in modern biomedicine. In this study, electrospun chitosan/polyethylene oxide (CS/PEO) nanofibers incorporating *Centella asiatica* extract were developed and evaluated in vitro as potential wound-healing materials. Nanofibers were fabricated using various CS/PEO ratios, and the 1:2 *w/w* composition loaded with 1% extract was selected as the optimal formulation based on morphological homogeneity and processing efficiency. Comprehensive characterization demonstrated that the nanofiber matrix provided sustained release of asiaticosides over several days, fitting best with Hixson–Crowell and Higuchi kinetic models, suggesting a combined diffusion–erosion mechanism. Biological assays confirmed that the optimized formulation displayed strong antioxidant and anti-inflammatory activity, with synergistic effects observed between chitosan and *C. asiatica*. Moreover, chitosan contributed intrinsic antimicrobial properties against *Staphylococcus aureus* and *Klebsiella pneumoniae*, while the extract provided additional antioxidant and regenerative potential. Biocompatibility studies in human fibroblasts showed no cytotoxic effects, and scratch assays confirmed that extract-loaded nanofibers significantly accelerated wound closure compared to the control and CS/PEO base. Taken together, the results highlight the potential of CS/PEO nanofibers with *C. asiatica* extract as multifunctional wound dressings that integrate structural support, controlled release, antimicrobial protection, and regenerative bioactivity. Future work should address in vivo evaluation, scale-up of electrospinning, and potential incorporation of synergistic antimicrobial agents to further enhance clinical applicability. This approach underlines the value of combining natural product pharmacology with biopolymer engineering in the design of next-generation wound-healing biomaterials.

## 1. Introduction

Chronic and hard-to-heal wounds remain a significant clinical challenge worldwide, often resulting from impaired cellular response, persistent inflammation, or microbial infection [1]. Wound healing is a complex, non-linear process that involves overlapping stages of hemostasis, inflammation, proliferation, and remodeling, which can be significantly influenced by both extrinsic and intrinsic factors, such as cytokines, growth factors, and oxidative stress. Interruptions or imbalances in these stages often lead to delayed healing or chronic wounds [2]. Consequently, various strategies have been developed to accelerate and improve the healing process. Any choice of wound treatment strategy should take into account the development of bacterial resistance, which is important in the current post-antibiotic era. Treatment compliance should also be considered. Bifunctionality expressed by the selection of appropriate biopolymers and active compounds is an important procedure when developing effective and convenient solutions for wound healing.

Biopolymer-based materials have gained prominence in wound care because of their natural origin, biocompatibility, and capacity to mimic the extracellular matrix [3]. Among them, chitosan is particularly attractive owing to its polycationic nature, which imparts antimicrobial, antioxidant, and anti-inflammatory properties, while also enabling its use as a versatile drug delivery platform [4,5]. Chitosan has already found applications in wound dressings, membranes, and foils, supporting skin regeneration and accelerating the closure of burns, ulcers, and postoperative wounds. Furthermore, chitosan blended with polyethylene oxide (PEO) significantly improves electrospinnability and enables the formation of continuous nanofibers, which can serve as three-dimensional scaffolds to support tissue regeneration [6]. Electrospinning, based on the application of electrostatic forces, has emerged as a powerful technique to produce nanofibrous mats with high surface area, porosity, and tunable morphology, making them excellent candidates for biomedical applications [7].

Biopolymer-based materials can be effectively enhanced with natural additives, including plant-derived extracts exhibiting multifunctional biological activity. *Centella asiatica* is an excellent example of such an ingredient [8,9]. *Centella asiatica*, also known as Gotu kola, is a medicinal herb rich in pentacyclic triterpenoids, including asiaticoside, madecassoside, and madecassic acid, along with other constituents such as centellose and centelloside [10]. Among them, asiaticoside is considered the major bioactive compound in aqueous extracts of *C. asiatica* (CAE) and has been extensively applied in traditional and alternative medicine formulations. Various biological activities of asiaticoside have been reported, including inhibition of keratinocyte proliferation, induction of collagen synthesis, and suppression of pro-inflammatory cytokine and chemokine activity [11,12]. These effects highlight its therapeutic potential in modulating skin repair and regeneration.

The innovative aspect of this study lies in the combination of *C. asiatica* extract with chitosan/PEO nanofibers obtained by electrospinning. While chitosan-based nanofibers are well recognized for their structural and antimicrobial properties, and *C. asiatica* is widely known for its regenerative and anti-inflammatory potential [9], few studies have explored their integration into a single multifunctional wound-healing system [13,14,15]. Incorporating asiaticoside-rich extract into electrospun nanofibers not only enhances therapeutic potential but also allows for controlled release of bioactive compounds directly at the wound site, thus addressing the limitations of conventional formulations.

This study aims to develop and comprehensively characterize electrospun chitosan/PEO nanofibers loaded with *Centella asiatica* extract as a multifunctional wound-healing platform. The novelty of this work lies in integrating asiaticoside-rich phytochemicals with a hybrid CS/PEO nanofibrous matrix to achieve sustained release up to 7 days, which marks a significant improvement compared with our previously developed hydrogels and 3D-printed chitosan systems that released the extract within 24 h. By combining a natural bioactive extract with a biopolymer–synthetic polymer nanofiber scaffold, this study introduces a new approach to prolonged topical delivery of triterpenoid saponins and expands the therapeutic potential of electrospun wound dressings.

Compared with our previously developed Centella asiatica delivery platform, chitosan hydrogels [13] and 3D-printed chitosan-based scaffolds [14], the electrospun nanofiber system investigated in this work introduces a significant functional improvement. Both earlier systems provided rapid release of asiaticosides, with complete release occurring within approximately 24 h, which limits their applicability in chronic wound environments that require prolonged exposure to bioactive compounds. In contrast, the nanofiber matrix described here enables sustained asiaticoside release for up to 7 days, resulting from a combined diffusion–erosion mechanism. This extended release profile represents a substantial advancement over our earlier formulations and provides a clear rationale for developing a nanofibrous dressing as a novel dosage form. It is also worth noting that the developed nanofibers offer a completely different functional use compared to hydrogel and 3D-printed dressings. Nanofibers offer significantly improved comfort of use, eliminating abrasion and unwanted loss of the applied system due to its significantly greater thickness or viscosity.

## 2. Results and Discussion

The present study focused on the development of chitosan-based nanofibers incorporating *Centella asiatica* extract, with the aim of selecting the optimal extract concentration and polymer composition to achieve favorable physicochemical and biological properties. The combination of chitosan with polyethylene oxide (PEO) provided a stable and flexible polymeric matrix characterized by good rheological properties, enhanced water retention, and improved mechanical resistance. Importantly, the incorporation of natural bioactive compounds into such biopolymeric nanofibers is expected to reduce potential side effects while ensuring a controlled and sustained release profile, thereby supporting wound-healing applications.

The *Centella asiatica* extract was prepared under previously optimized conditions (70% methanol, 70 °C, three ultrasonic extraction cycles of 60 min each), followed by freeze-drying [13].

In the first stage, the influence of polymer composition on the electrospinning process was evaluated. Pure chitosan exhibits poor spinnability due to its high viscosity, limited chain entanglement, and strong intermolecular interactions, which hinder the formation of continuous and defect-free fibers [16]. To overcome these limitations, polyethylene oxide (PEO) was incorporated as a co-polymer. PEO is a synthetic, water-soluble polymer with excellent electrospinnability, high molecular flexibility, and the ability to form uniform nanofibers [17]. When blended with chitosan, PEO improves solution viscosity, enhances chain entanglement, and reduces electrostatic repulsion between chitosan’s polycationic groups. As a result, the addition of PEO facilitates stable jet formation during electrospinning and contributes to the production of continuous nanofibers with improved morphology and mechanical stability [18].

**Figure 1:**
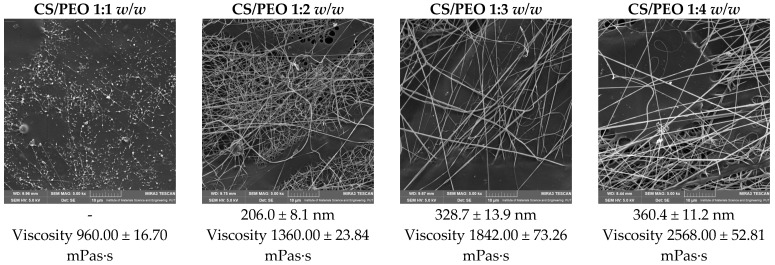
SEM images of nanofibers and their diameters, and solution viscosity before electrospinning.

Figure 1 presents SEM micrographs of chitosan/PEO nanofibers obtained at different polymer ratios (CS/PEO = 1:1, 1:2, 1:3, and 1:4 *w/w*). Distinct differences in morphology and fiber diameter can be observed depending on the proportion of PEO. At the 1:1 ratio, the fibers exhibit morphological defects in the form of bead-like structures, indicating insufficient spinnability and reduced homogeneity of the material. Increasing the proportion of PEO results in the formation of smoother, more continuous nanofibers with higher uniformity. This improvement can be attributed to the enhanced chain entanglement and viscoelastic properties of the polymer solution. Quantitative analysis of fiber diameters confirmed that the average diameter increased with rising PEO content, ranging from 206.0 ± 80.5 nm at 1:2 to 360.4 ± 111.8 nm at 1:3 and 328.7 ± 139.5 nm at 1:4. These results demonstrate that the addition of PEO significantly influences nanofiber morphology, with the CS/PEO 1:2 and 1:3 systems exhibiting the most favorable structural properties for further functionalization. This also highlights the essential role of PEO as a synthetic co-spinning polymer that enables the electroprocessing of the natural biopolymer chitosan; therefore, the final nanofiber matrix should be regarded as a hybrid system composed of both natural and synthetic polymers rather than a purely biopolymer-based material.

As shown by the viscosity values included in Figure 1, increasing the PEO proportion leads to a progressive rise in solution viscosity, which directly improves chain entanglement and stabilizes jet formation during electrospinning. The viscosity values obtained for the CS/PEO systems fall within the range typically reported as suitable for stable electrospinning (approximately 100–3000 mPa·s), ensuring sufficient chain entanglement and jet continuity during fiber formation [19].

Several studies have confirmed that PEO content directly influences nanofiber morphology and diameter. Szymańska et al. reported that chitosan/PEO mats in a 1:4 ratio yielded nanofibers with an average diameter of 266 ± 114 nm, highlighting the stabilizing role of PEO in electrospinning and its impact on drug release profiles [20]. Similarly, Liu et al. demonstrated that blends containing equal proportions of chitosan and PEO produced smooth and homogeneous nanofibers with diameters of 53.93 ± 17.07 nm [21], while Murillo et al. obtained fibers of 124 ± 36 nm using a CS/PEO 1:1 system [22]. These findings indicate that increasing PEO concentration generally results in larger fiber diameters and reduced bead formation, improving fiber homogeneity and mechanical stability. Collectively, the literature evidence confirms that the presence of PEO is crucial for achieving defect-free chitosan nanofibers, while its proportion enables tuning of fiber diameter and structural features depending on the intended biomedical application.

**Figure 2:**
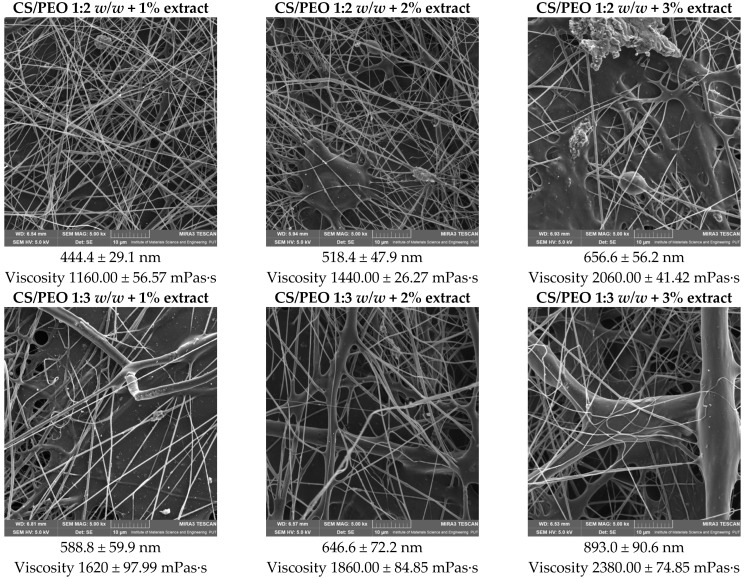
SEM images of nanofibers with extract and their diameters, and solution viscosity before electrospinning.

In the second stage of the study, the *Centella asiatica* extract was incorporated into the selected hydrogel bases at concentrations of 1%, 2%, and 3% (*w/w*). The objective was to evaluate how the increasing extract content influences the morphology and homogeneity of the obtained nanofibers. SEM analysis confirmed that the addition of the extract affected both the structural integrity and the diameter of the fibers. Figure 2 presents SEM micrographs of CS/PEO nanofibers (ratios 1:2 and 1:3 *w/w*) loaded with varying concentrations of the extract. At a concentration of 1%, the nanofibers maintained good continuity and uniform morphology, with diameters ranging between ~444 nm and ~589 nm, depending on the polymer ratio. However, higher extract concentrations (2% and 3%) resulted in a progressive increase in average fiber diameter (up to ~893 nm in CS/PEO 1:3 with 3% extract) and reduced homogeneity, as evidenced by the appearance of irregularities and bead-like structures. This effect can be attributed to changes in the viscosity and conductivity of the polymer solution, which disrupts the stability of the electrospinning jet. Taken together, the results demonstrate that moderate extract loading (1%) is most favorable for obtaining uniform and defect-free nanofibers, while higher concentrations compromise fiber quality. These findings suggest that polymer–extract interactions and solution parameters must be carefully balanced to optimize the electrospinning process and produce nanofibers with desirable morphological features.

So, for all subsequent experiments, the CS/PEO (1:2 *w/w*) nanofibers containing 1% *Centella asiatica* extract were selected as the optimal formulation and used for further analyses.

**Figure 3:**
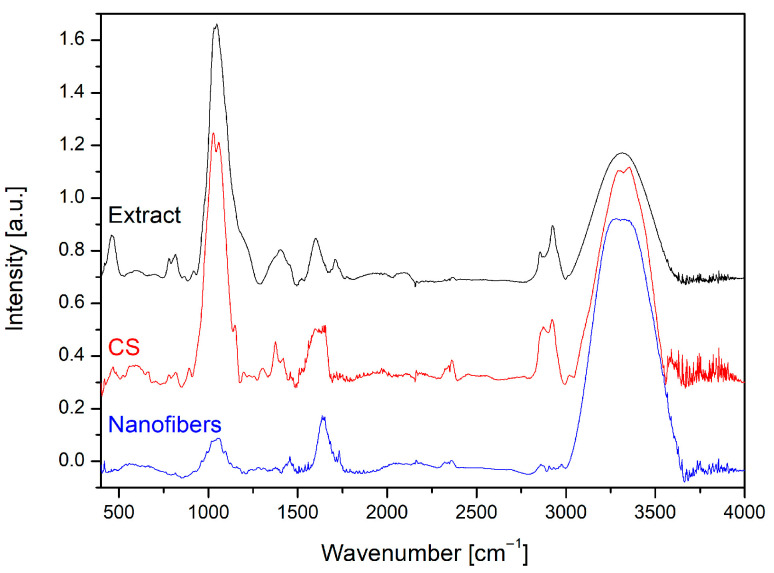
FTIR spectra of *Centella asiatica* extract, CS LMW, and nanofibers with extract.

The FTIR spectra of *Centella asiatica* extract and chitosan have been previously described in detail [13,14], and the characteristic bands corresponding to asiaticoside and CS were confirmed in nanofiber samples. Importantly, in the spectra of the composite nanofibers, no new absorption peaks were observed (Figure 3), only shifts and overlapping of the characteristic bands. In addition to the signals originating from chitosan and the extract, the spectrum also contained dominant PEO-related bands, particularly the strong C–O–C stretching vibration at ~1100 cm^−1^, as well as the CH_2_ stretching bands at ~2880–2930 cm^−1^ and the CH_2_ rocking vibration near 840–950 cm^−1^, which are typical for polyethylene oxide [23]. The most noticeable changes were small shifts and broadening within the O–H/N–H stretching region around 3350–3300 cm^−1^, characteristic of hydroxyl and amine groups, indicating stronger hydrogen bonding between the extract and the CS/PEO matrix. In addition, the amide I (C=O stretching) band of chitosan at ~1640 cm^−1^ and the amide II (N–H bending) band near ~1580 cm^−1^ partially overlapped with the asiaticoside carbonyl band at ~1730–1720 cm^−1^. Overlapping and broadening of the C–O–C asymmetric stretching (~1150 cm^−1^) and C–O stretching of secondary alcohols (~1060–1050 cm^−1^) reflected contributions from both the polysaccharide backbone of chitosan and the glycosidic structure of asiaticoside, in addition to the strong ether bands of PEO. This overall pattern confirms that the components interact through physical interactions, mainly hydrogen bonding, rather than forming new covalent bonds, allowing the nanofibers to preserve the structural integrity and bioactivity of the incorporated extract.

**Figure 4:**
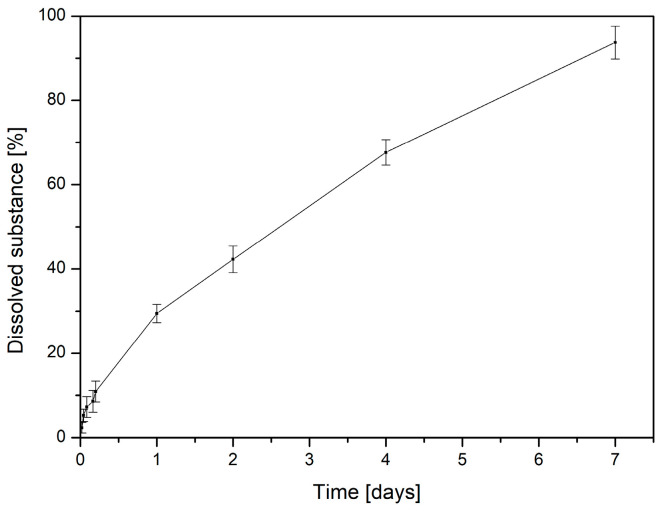
Release profile of asiaticoside from nanofibers. Data are expressed as mean ± SD (n = 3). Error bars represent standard deviations. The asiaticoside content was 4.00 ± 0.02 µg per 100 mg of nanofibers, quantified as 100% of the reference standard.

For a formulation to exert its therapeutic effect, it must ensure the efficient release of incorporated bioactive compounds. In this study, the in vitro release of asiaticosides from CS/PEO (1:2 *w/w*) nanofibers loaded with *Centella asiatica* (1%) was analyzed using common kinetic models (zero-order, first-order, Higuchi, Korsmeyer–Peppas, and Hixson–Crowell) (Figure 4, Table S1 in Supplementary Materials). As shown in Table 1 and Figure 4, the dataset is best described by the Hixson–Crowell cube-root model (highest R^2^), indicating that release is governed by changes in the effective surface area/geometry of the matrix rather than by simple diffusion alone. The Higuchi model also provides an excellent description, supporting a diffusion-controlled component at early and intermediate times. The Korsmeyer–Peppas analysis (log–log linearization) yields an exponent n typical of anomalous (non-Fickian) transport, consistent with a combined mechanism in which diffusion from the hydrated polymer network is coupled with matrix relaxation/erosion. Zero-order and first-order descriptions are less adequate for this system.

To gain deeper insight into the release mechanism, the dissolution profile was analyzed using the Peppas–Sahlin model [24]. First, the Korsmeyer–Peppas equation was fitted to the early stage of release (≤60% of drug released), and the kinetic exponent n was calculated from the slope of the log–log plot of $ln(M_{t}/M_{\infty })$ versus lnt. The obtained value of n indicated a non-Fickian transport regime. The value of m in the Peppas–Sahlin equation was therefore fixed to n, and the two model constants ($k_{1}$ and $k_{2}$) were determined by linear regression. The coefficient $k_{1}$ describes the contribution of Fickian diffusion, whereas $k_{2}$ reflects the relaxation-(or erosion-) driven transport. The fitted model showed excellent agreement with the experimental data (R^2^ close to 1), confirming the suitability of the Peppas–Sahlin approach for this formulation. Positive values of both constants indicated that both mechanisms participated in the release process. However, $k_{1}$ was higher than $k_{2}$, and the ratio R/F remained below 1 over the whole-time interval, suggesting that Fickian diffusion was the predominant mechanism. The simulated contributions further confirmed that the diffusion term $k_{1}t^{m}$ dominated especially at shorter release times, while the relaxation component $k_{2}t^{2m}$ increased gradually with time. These results indicate that the drug is released mainly by diffusion through the hydrated polymeric matrix, while polymer relaxation becomes more relevant at later stages.

Mechanistically, this behavior is consistent with gradual hydration and swelling of the CS/PEO network [25]. In the release medium at pH 5.5, PEO, as a highly water-soluble polymer, dissolves rapidly, leading to an initial loss of mass and contributing to the early-stage burst release and erosion of the nanofiber surface. In contrast, chitosan undergoes gradual hydration, protonation, and swelling, forming a more stable but progressively loosening network. Therefore, the release profile likely reflects a two-step process in which the rapid dissolution of PEO governs the initial phase, while the subsequent swelling and partial erosion of chitosan sustain the later stages of asiaticoside release. This behavior is consistent with the strong fit of the Hixson–Crowell model, which accounts for geometry changes, and with the mixed diffusion–relaxation mechanism indicated by the Korsmeyer–Peppas and Peppas–Sahlin analyses. Such a multi-stage, geometry-affected process is advantageous for wound-care applications, where prolonged exposure to triterpenoid saponins supports ongoing antioxidant and anti-inflammatory action at the wound bed. This behavior is particularly advantageous in wound-healing applications, where prolonged availability of active compounds is desirable to ensure continuous support for tissue regeneration and protection against oxidative and inflammatory damage [26].

**Figure 5:**
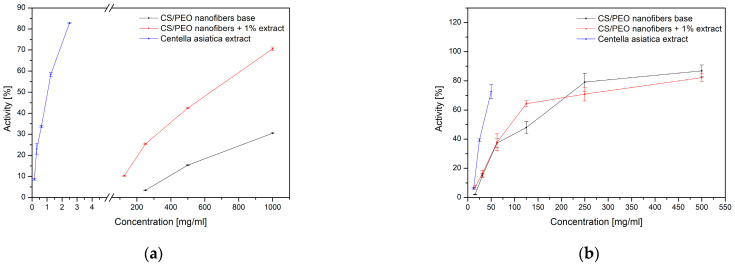
Concentration–response curves for antioxidant (**a**) and anti-inflammatory (**b**) activities.

The biological activity of the obtained nanofibers was assessed in terms of antioxidant and anti-inflammatory properties (Figure 5, Table 2). The base CS/PEO (1:2 *w/w*) system exhibited only moderate antioxidant potential, whereas the incorporation of *Centella asiatica* extract significantly enhanced radical scavenging capacity. The comparison with the extract alone suggests that although the polymer matrix may limit the immediate availability of active compounds, it provides stabilization and controlled release, resulting in improved overall antioxidant performance. The sustained release profile of asiaticosides from the nanofiber matrix directly contributes to the prolonged antioxidant and anti-inflammatory activity observed in vitro. Unlike the free extract, which provides rapid but transient bioactivity, the controlled release from the CS/PEO nanofibers ensures continuous availability of triterpenoid saponins over time. This release-driven exposure likely underlies the enhanced and more stable inhibition of DPPH radicals and hyaluronidase activity, highlighting the functional advantage of the nanofibrous delivery system over conventional formulations.

A similar tendency was observed for anti-inflammatory activity evaluated through hyaluronidase inhibition (Table 2). While the extract alone showed only weak inhibition, the nanofiber formulation containing *Centella asiatica* displayed markedly stronger effects. Notably, even the CS/PEO base demonstrated measurable inhibition, confirming the intrinsic bioactivity of chitosan. These findings indicate a synergistic effect between the natural extract and the chitosan-based carrier, which not only enhances antioxidant and anti-inflammatory properties but also supports the potential of the developed nanofibers as multifunctional biomaterials for wound-healing applications.

The enhanced activity observed for the composite system is consistent with the pharmacological properties of *C. asiatica* triterpenoid saponins, such as asiaticoside and madecassoside. These compounds act as direct radical scavengers and modulate cellular responses by downregulating pro-inflammatory cytokines (TNF-α, IL-1β, and IL-6) and promoting fibroblast proliferation and collagen synthesis [10]. At the same time, the inhibitory effect of chitosan on hyaluronidase activity can be attributed to several complementary mechanisms. As a polycationic biopolymer, chitosan possesses protonated amino groups that can interact electrostatically with negatively charged residues of the enzyme and with hyaluronic acid, thereby hindering substrate access to the catalytic site [27]. Furthermore, the formation of dense polymeric matrices, such as nanofibers or hydrogels, provides a physical diffusion barrier that restricts the penetration of hyaluronic acid to the enzyme [28]. Chitosan has also been reported to immobilize enzymes on its surface through ionic and hydrogen bonding, which reduces their catalytic mobility and overall enzymatic efficiency [29]. The synergistic effect observed in our study reflects these complementary mechanisms. Similar findings were reported by Phupaisan et al., who demonstrated improved anti-hyaluronidase activity when *C. asiatica* was combined with other antioxidants [30], and by Paczkowska-Walendowska et al., who showed that chitosan scaffolds enriched with baicalein-rich extracts enhanced both antioxidant and anti-inflammatory potential [31].

The antimicrobial potential of the obtained nanofibers was evaluated against representative Gram-positive (*Staphylococcus aureus*), Gram-negative (*Klebsiella pneumoniae*), and fungal (*Candida albicans*) strains (Table 3). These microorganisms were selected as clinically relevant models because they are among the most common pathogens associated with skin and soft tissue infections as well as chronic wound colonization. *S. aureus* is a leading cause of wound infections, known for its ability to form biofilms and secrete toxins that impair tissue healing [32]. *K. pneumoniae*, a Gram-negative opportunistic pathogen, is increasingly associated with multidrug-resistant wound infections, where it exacerbates inflammation and delays regeneration [33]. While *C. albicans* represents fungal pathogens frequently isolated from chronic wounds, its capacity to adhere, form biofilms, and secrete hydrolytic enzymes contributes to persistent infection and impaired tissue repair [34]. The CS/PEO nanofiber base demonstrated the strongest activity, particularly against *S. aureus* and *K. pneumoniae*, with low minimum inhibitory concentrations (MICs). In contrast, the extract-loaded nanofibers exhibited weaker antimicrobial effects, and the *C. asiatica* extract alone showed negligible activity within the tested concentration range. These results suggest that the antimicrobial effect is mainly attributed to the chitosan component, while the incorporation of the plant extract may dilute or partially mask this activity. These observations clearly indicate that the antimicrobial properties of the nanofiber system are primarily derived from the chitosan component. Chitosan is well known for its broad-spectrum antimicrobial properties, which result from its polycationic nature. The positively charged amino groups interact with negatively charged bacterial membranes, leading to increased permeability, leakage of intracellular constituents, and ultimately cell death [35]. Additionally, chitosan can chelate essential metal ions and bind to microbial DNA, further impairing microbial metabolism and replication [36]. This explains the pronounced antibacterial activity of the nanofiber base observed in our study. The reduced antimicrobial activity of the extract-loaded nanofibers compared to the CS/PEO base can be attributed to several factors. Incorporation of the C. asiatica extract likely results in partial coating of the chitosan surface and masking of its protonated –NH_3_^+^ groups, thereby limiting the electrostatic interactions responsible for membrane disruption in bacteria. In addition, the extract modifies fiber morphology and increases fiber diameter, which reduces the effective surface area of chitosan available for antimicrobial action. These combined effects explain the higher MIC values observed for the composite formulation. Previous studies have similarly demonstrated that combining *C. asiatica* with other bioactive agents often requires optimization to balance wound-healing benefits with antimicrobial protection [13].

**Figure 6:**
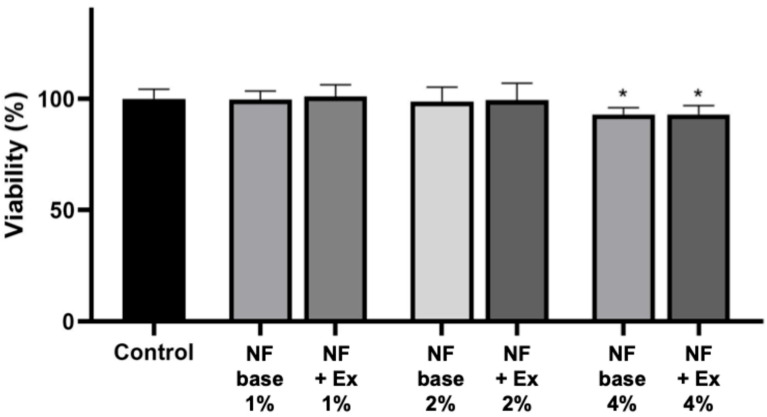
In vitro biocompatibility of CS/PEO nanofibers evaluated on human skin fibroblasts. NF Base—CS/PEO nanofiber base, NF + Ex—CS/PEO nanofibers loaded with *Centella asiatica* extract. (*) *p* < 0.05.

Biocompatibility of nanofibers was evaluated using human skin fibroblasts, and the results are presented in Figure 6. Both the CS/PEO base and the extract-loaded nanofibers maintained high cell viability, comparable to the untreated control. No cytotoxic effects were observed, indicating that the developed nanofibers are well tolerated by fibroblasts. Interestingly, cells exposed to the extract-containing formulation showed slightly improved metabolic activity compared to the base, suggesting a potential stimulatory effect of *Centella asiatica* constituents on fibroblast proliferation. These findings are consistent with previous reports demonstrating the favorable biocompatibility of chitosan-based nanofibers and the regenerative potential of *C. asiatica* extract in wound-healing models [13,14]. The results confirm that the obtained nanofibers are suitable for biomedical use, particularly in wound dressings where cytocompatibility is a prerequisite for clinical application.

**Figure 7:**
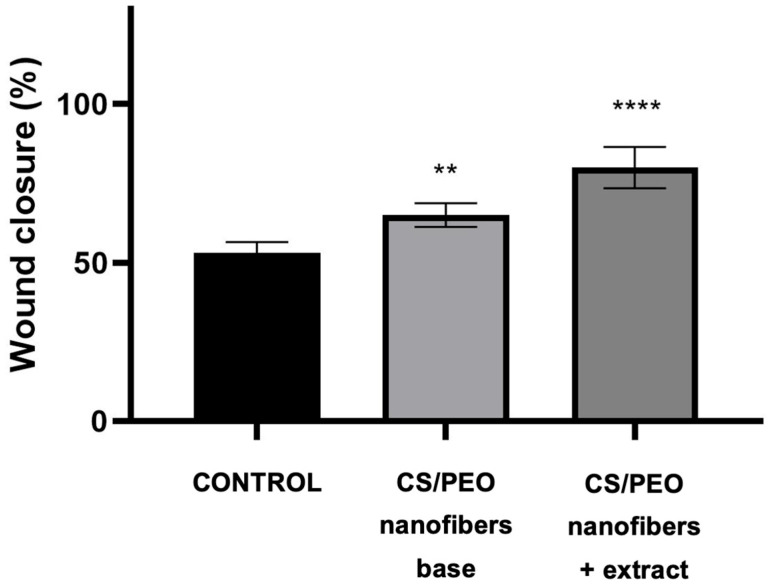
Effect of nanofibers on post-scratch wound closure by human normal skin fibroblasts (Hs27 cells). Results are expressed as means ± SD. Statistical significance was designated as (**) *p* < 0.01; (****) *p* < 0.001 (vs. control at the respective time points) using a two-way ANOVA followed by Tukey’s post hoc test.

**Figure 8:**
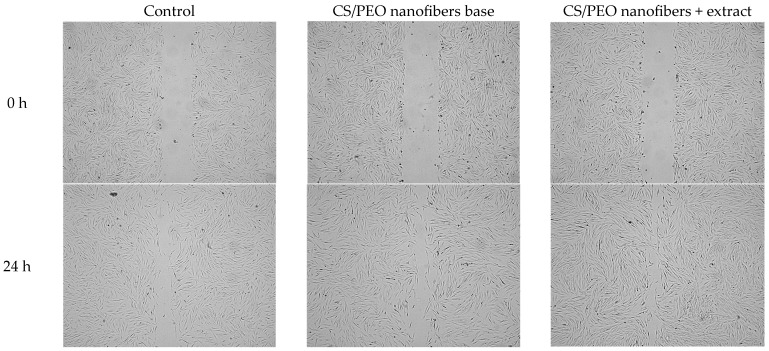
Representative images of wound-healing properties of control, nanofiber base, and combination of nanofiber with *Centella asiatica* extract.

The wound-healing potential of the developed nanofibers was further evaluated using a scratch assay in human skin fibroblasts (Hs27). As shown in Figure 7, cells treated with the extract-loaded nanofibers demonstrated significantly faster wound closure compared to both the untreated control and the chitosan/PEO base. Already after 24 h, nanofibers exhibited markedly reduced scratch width, indicating combined effects of enhanced fibroblast migration and proliferation. In contrast, the base formulation promoted moderate closure, confirming the intrinsic stimulatory effect of chitosan on cell migration. Representative microscopic images presented in Figure 8 illustrate these findings, showing more advanced wound closure in cultures treated with extract-containing nanofibers compared to control and base samples. This result highlights the synergistic role of *Centella asiatica* triterpenoid saponins, known to stimulate fibroblast proliferation, collagen synthesis, and angiogenesis, in combination with the bioactive chitosan carrier. Together, these effects support the potential of the developed nanofiber system as a bioactive wound dressing capable of accelerating tissue repair.

The enhanced wound closure observed in the scratch assay can be attributed to the combined effects of nanofiber architecture and sustained bioactive release. The high surface area and ECM-mimicking fibrous structure promote close cell–material interactions, facilitating fibroblast adhesion and migration. Simultaneously, the gradual release of Centella asiatica triterpenoids supports fibroblast proliferation and regenerative signaling. This synergistic interaction between material structure and biological activity explains the significantly faster wound closure achieved by the extract-loaded nanofibers compared to the polymer base and control.

The present nanofiber formulation provides several clear advantages over our previously reported delivery systems based on chitosan hydrogels and 3D-printed scaffolds. Most importantly, the nanofibers ensured sustained release of asiaticoside for up to 7 days, whereas both hydrogels [13] and 3D-printed constructs [14] exhibited complete release within 24 h. This difference results from the highly porous but slowly eroding electrospun matrix, which follows Hixson–Crowell and Higuchi kinetics. Prolonged release is particularly relevant in chronic wound management, where continuous exposure to triterpenoid saponins supports antioxidant, anti-inflammatory, and pro-regenerative processes. Furthermore, the nanofibers demonstrated enhanced anti-inflammatory activity and promoted faster fibroblast migration compared to the earlier formulations, likely due to higher surface area and more intimate cell–material interactions. These improvements confirm that electrospinning provides a significantly more effective platform for long-term delivery of asiaticoside-rich extracts. For future applications, incorporation of additional antimicrobial agents (e.g., silver nanoparticles, essential oils, or synergistic phytochemicals) may further enhance the spectrum of antimicrobial protection without compromising the beneficial effects of *C. asiatica*.

The presented solution of utilizing the therapeutic properties of *Centella asiatica* extract in wound healing through the use of a nanofiber matrix is an innovative approach that requires further research. Although, to our knowledge, this is the first report in this area of development, and although the developed CS/PEO nanofibers loaded with Centella asiatica extract demonstrated promising release characteristics and biological activity profiles, several limitations of the present study should be acknowledged. First, key material properties essential for wound-dressing applications, such as mechanical parameters (tensile strength, Young’s modulus, elongation at break) in both dry and hydrated states, were not evaluated. Similarly, wet-state performance metrics, including swelling behavior, water-uptake capacity, and water vapor transmission rate, were not determined, limiting the ability to fully assess moisture-management functionality. Future work should include these analyses to strengthen the understanding of structure–property relationships and to more comprehensively validate the suitability of the nanofiber system as a wound-dressing material.

In accordance with the principles of biomaterials development, comprehensive in vitro characterization represents a critical prerequisite for subsequent in vivo studies. At this stage, the present work was intentionally designed as an in vitro proof-of-concept to establish structure–function–bioactivity relationships prior to animal testing.

## 3. Materials and Methods

### 3.1. Plant Material

Plant material, *Centellaeasiaticaeherba*, was purchased from NANGA (Blękwit, Poland).

### 3.2. Chemicals and Reagents

Excipients, such as chitosan (low molecular weight = LMW 20–300 cps, 1% in 1% acetic acid) and polyethylene oxide, were supplied from Sigma-Aldrich (Poznan, Poland). Reagents for activity assays (2,2-Diphenyl-1-picrylhydrazyl (DPPH), sodium chloride, bovine serum (BSA), hexadecyltrimethylammonium bromide (CTAB), hyaluronic acid (HA), and hyaluronidase enzyme) and for dissolution studies (phosphate buffer) were obtained from Sigma-Aldrich (Poznan, Poland). The hydration solution was obtained from Pion Inc. (Billerica, MA, USA). High-quality pure water and ultra-high-quality pure water were prepared using a Direct-Q 3 UV Merck Millipore purification system (Merck Millipore, Darmstadt, Germany).

### 3.3. Preparation of Centella asiatica Extract

The extract was prepared according to previously optimized parameters [13]: solvent: 70% methanol, temperature: 70 °C, ultrasonic extraction duration: three cycles of 60 min each. It was then freeze-dried to obtain a dry form, which was used for further research. The optimized freeze-dried extract was standardized using the HPLC method, and the contents of asiaticoside (AS), asiatic acid (AA), and madecassic acid (MA) were obtained as 390.96 ± 2.24 µg/100 mg, 18.50 ± 0.08 µg/100 mg, and 3.89 ± 0.02 µg/100 mg lyophilized extract, respectively (sum of AS, AA, and MA: 413.35 ± 2.34 µg/100 mg lyophilized extract).

### 3.4. Preparations of Nanofibers

#### 3.4.1. Selection of the Nanofibers Base

Chitosan (CS) and polyethylene oxide (PEO) were selected as the polymeric components for electrospinning. Due to the limited spinnability of pure CS, PEO was used as a co-spinning agent to improve solution viscosity, chain entanglement, and jet stability during electrospinning.

Polymer solutions were prepared by dissolving CS in 2% (*v/v*) acetic acid under magnetic stirring until complete solubilization (Table 4). Separately, PEO was dissolved in distilled water. The solutions were then mixed at different weight ratios of CS to PEO (1:1, 1:2, 1:3, and 1:4 *w/w*) to obtain precursor solutions with varying polymer compositions. Homogenization was carried out under continuous stirring for 12 h at room temperature until clear and uniform blends were obtained.

The viscosity values of solutions were evaluated at a shear rate of 100 rpm using a 07 spindle Brookfield DV2T viscometer (Brookfield, Middleboro, MA, USA) at 25 °C, assessed using Rheocalc T software (version 1.2.19). All the experiments were performed in triplicate.

The electrospinning process was performed using the NS + NanoSpinner Plus Electrospinning Equipment (Inovenso Ltd., Istanbul, Turkey). The polymer solutions were loaded into 5 mL syringes fitted with stainless steel needles (internal diameter 0.8 mm). Electrospinning was carried out at an applied voltage of 27 kV, a flow rate of 2 mL/h, and a tip-to-collector distance of 12 cm. The nanofibers were collected on aluminum foil and dried for 24 h to remove residual solvent.

#### 3.4.2. Selection of the Composition of Nanofibers Containing the Extract

The previously prepared extract was incorporated into the selected CS/PEO blends (1:2 and 1:3 *w/w*) at concentrations of 1%, 2%, and 3% (*w/w*).

#### 3.4.3. Scanning Electron Microscopy (SEM)

The surface morphology of the nanofibers was examined by scanning electron microscopy (SEM) using a Quanta 250 FEG instrument (Thermo Fisher Scientific, Waltham, MA, USA). Prior to imaging, the samples were sputter-coated with a thin layer of gold–palladium to enhance conductivity. The fiber diameters were determined from the obtained micrographs using ImageJ analysis software (version 1.54).

#### 3.4.4. Fourier Transform Infrared Spectroscopy (FTIR)

FTIR spectra were recorded in absorbance mode using an IRTracer-100 spectrophotometer (Shimadzu, Kyoto, Japan) equipped with LabSolutions IR software (v.1.86 SP2). Measurements were carried out in the 400–4000 cm^−1^ range with a resolution of 4 cm^−1^, employing Happ–Genzel apodization and averaging 400 scans.

#### 3.4.5. Dissolution Behavior

Dissolution studies of electrospun nanofibers were performed using an Agilent 708-DS (Agilent, Santa Clara, CA, USA) apparatus equipped with the conventional basket method at 37 ± 0.5 °C and 50 rpm. The nanofibers were immersed in 30 mL of phosphate buffer (pH 5.5). At predefined time intervals, samples of the acceptor medium were collected and filtered through a 0.45 µm nylon membrane filter. The concentrations of asiaticoside were determined using a previously validated HPLC method [13]. All experiments were conducted in triplicate.

pH 5.5 was selected because it closely mimics the slightly acidic environment of healthy human skin (pH 4.7–5.6) as well as the microenvironment of the wound bed, which typically becomes mildly acidic during the early inflammatory and proliferative phases of healing. Such conditions directly influence chitosan swelling, protonation of amino groups, and polymer relaxation, thereby providing a more physiologically relevant simulation of how the nanofiber matrix would behave upon contact with the skin surface or a healing wound.

### 3.5. Determination of Nanofibers Biological Activity

#### 3.5.1. Antioxidant Activity

The antioxidant activity was assessed by the 2,2-diphenyl-1-picrylhydrazyl (DPPH) radical scavenging assay, following a previously described procedure [27]. Briefly, a volume of 25 μL of each extract was mixed with 175 μL of a DPPH solution (3.9 mg dissolved in 50 mL of methanol). The mixtures were vortexed and allowed to incubate for 30 min at room temperature in the dark. As a control, the absorbance of 25 μL of water combined with 175 μL of methanol was measured at 517 nm against a blank composed of 25 μL of water combined with 175 μL of DPPH solution. Each measurement was performed in nine replicates. The ascorbic acid was used as the positive control.

#### 3.5.2. Anti-Inflammatory Activity

The hyaluronidase inhibition was evaluated using a turbidimetric method previously established in the literature [27]. Briefly, a mixture consisting of 25 µL of hyaluronidase solution (30 U/mL in acetate buffer, pH 7.0), 25 µL of acetate buffer (50 mM, pH 7.0, containing 77 mM NaCl and 1 mg/mL albumin), 15 µL of acetate buffer (pH 4.5), and 10 µL of the samples was prepared and incubated at 37 °C for 10 min. Subsequently, 25 µL of hyaluronic acid solution (0.3 mg/mL in acetate buffer, pH 4.5) was added, and the mixture was further incubated at 37 °C for 45 min. To precipitate the undigested hyaluronic acid, 200 µL of 2.5% CTAB in 2% NaOH (pH 12) was added, followed by incubation at room temperature for 10 min. The turbidity of the resulting mixture was then measured at 600 nm. Each measurement was performed in six replicates. The β-escin was used as the positive control.

#### 3.5.3. Microbiological Activity

The minimal inhibitory concentrations (MICs) were determined using the microdilution method. Bacterial cultures were grown in tryptic soy broth (TSB, Graso Biotech, Starogard Gdański, Poland) in 96-well microplates (Nest Scientific Biotechnology, Wuxi, Jiangsu, China) with a final volume of 100 µL per well, ensuring an inoculum density of 10^6^ CFU/mL. The bacterial suspensions were standardized according to the McFarland scale. Serial dilutions of each hydrogel were prepared, starting from an initial concentration of 20 mg/mL. Additional methodological details can be found in our earlier publication [37]. The plates were incubated for 24 h at 36 °C, after which MIC values were determined either by visual assessment or by using a colorimetric reaction following the addition of 10 µL of a 1% aqueous solution of 2,3,5-triphenyl tetrazolium chloride (TTC) (Sigma Aldrich, Poznań, Poland). Each measurement was performed in three replicates

#### 3.5.4. Biocompatibility

The viability of human normal skin fibroblasts (Hs27 cells) incubated for 24 h with nanofibers was examined using the MTT assay [31]. Briefly, Hs27 fibroblasts were seeded into 96-well plates at a density of 1 × 10^5^ cells/mL and allowed to attach for 24 h. The medium was then replaced with DMEM containing 2% FBS and the respective test samples, while control cells received only the medium. After 24 h of incubation, cytotoxicity was assessed using the MTT assay. Cells were washed with PBS and treated with 100 µL of medium containing 10% MTT solution (5 mg/mL), followed by a 4 h incubation at 37 °C. Subsequently, 100 µL of 10% SDS solution was added to dissolve the resulting formazan crystals, and absorbance was measured at 570 nm after overnight incubation. All experiments were performed twice, each in triplicate.

#### 3.5.5. Wound-Healing Properties

The wound-healing potential of the nanofibers was evaluated on Hs27 cells using the scratch assay, following the previously described method [31]. Cells were cultured in high-glucose DMEM supplemented with 10% FBS and antibiotics, then seeded in 6-well plates at 1 × 10^5^ cells/mL. Once the monolayer reached ~90% confluence, a linear scratch was created using a sterile pipette tip. After washing the wells with PBS to remove debris, fresh medium containing the tested samples or 2% FBS (control) was added. Images of the scratch area were captured at 0 and 24 h using an Olympus CKX53 microscope (Olympus, Tokyo, Japan). The assay was performed in triplicate (n = 3), and statistical significance was evaluated using two-way ANOVA with treatment and time as factors, followed by Tukey’s post hoc test. The significance thresholds used were: *p* < 0.05 (*), *p* < 0.01 (**), *p* < 0.001 (***), and *p* < 0.0001 (****), as indicated in the figure caption.

## 4. Conclusions

In this study, chitosan/PEO nanofibers incorporating *Centella asiatica* extract were successfully developed and characterized as multifunctional wound-healing materials. The optimized formulation (CS/PEO 1:2 *w/w* with 1% extract) exhibited favorable morphological features, sustained release of asiaticoside, potent antioxidant and anti-inflammatory activities, intrinsic antimicrobial effects from chitosan, excellent biocompatibility, and the ability to accelerate fibroblast migration in vitro.

Taken together, these results indicate that the designed nanofibers can serve as a promising platform for advanced wound dressings, combining structural support with bioactive functionality. Importantly, the work highlights the synergistic contribution of chitosan and *C. asiatica* compounds, which together provide a dual mode of action, protecting against oxidative and inflammatory damage while promoting tissue regeneration.

From a translational perspective, future studies should focus on in vivo validation of the wound-healing efficacy, long-term biocompatibility, and integration of additional antimicrobial agents to strengthen protection against multidrug-resistant pathogens. The presented in vitro dataset provides a necessary and ethically responsible foundation for future in vivo evaluation, minimizing unnecessary animal use in accordance with the 3R principles. Moreover, tailoring the electrospinning process toward scalable production and exploring 3D-printed or hybrid biomaterials may open new avenues for clinical application. Ultimately, this approach provides a foundation for the development of next-generation, multifunctional wound dressings that bridge natural product pharmacology with modern biomaterial engineering.

## Figures and Tables

**Table 1:** Kinetic modeling of asiaticoside release from CS/PEO nanofibers.

Zero-Order		First-Order		Higuchi		Korsmeyer–Peppas		Hixson–Crowell		Peppas–Sahlin	
Rate Constant	R^2^	Rate Constant	R^2^	Rate Constant	R^2^	Release Exponent	R^2^	Rate Constant	R^2^	Parameters	R^2^
k_0_ = 13.15	0.9675	k_1_ = 0.37	0.9700	k_H_ = 36.05	0.9948	n = 0.60	0.9884	k_HC_ = 0.38	0.9953	n = 0.52 m = 0.52 k_1_ = 0.43 k_2_ = 0.10	0.9930

**Table 2:** Antioxidant and anti-inflammatory activities of nanofibers.

	Antioxidant Activity Inhibition of the DPPH Radical Activity [%]	Anti-Inflammatory Activity Inhibition of the Activity of the Enzyme Hyaluronidase [IC_50_]
CS/PEO nanofibers base	30.47 ± 0.30	136.83 ± 25.71 mg/mL
CS/PEO nanofibers + 1% extract	70.62 ± 0.84	95.24 ± 23.74 mg/mL
*Centella asiatica* extract	IC_50_ = 1.04 ± 0.02 mg/mL	34.67 ± 4.67 mg/mL
Ascorbic acid	IC_50_ = 0.066 mg/mL	-
β-escin	-	0.74 ± 0.01 mg/mL

**Table 3:** MIC values of nanofibers and extract against selected wound pathogens.

	*Staphylococcus aureus*	*Klebsiella pneumoniae*	*Candida albicans*
CS/PEO nanofibers base	1.56 mg/mL	3.125 mg/mL	25 mg/mL
CS/PEO nanofibers + 1% extract	12.5 mg/mL	12.5 mg/mL	25 mg/mL
*Centella asiatica* extract	>50 mg/mL	>50 mg/mL	>50 mg/mL

**Table 4:** Composition of CS/PEO systems.

	CS/PEO 1:1 *w/w*	CS/PEO 1:2 *w/w*	CS/PEO 1:3 *w/w*	CS/PEO 1:4 *w/w*
CS/PEO weight ratio *w/w*	1:1	1:2	1:3	1:4
H_2_O (mL)	20	20	20	20
Chitosan (g)	0.4	0.4	0.4	0.4
PEO (g)	0.4	0.8	1.2	1.6
Acetic acid (mL)	0.2	0.2	0.2	0.2
Methanol (mL)	20	20	20	20

## Data Availability

The original contributions presented in this study are included in the article/Supplementary Material. Further inquiries can be directed to the corresponding author.

## Supplementary Materials

The following supporting information can be downloaded at: https://www.mdpi.com/article/10.3390/ijms262412134/s1.
