# Supplementary material for: Chitosan/PEO Nanofibers as a Delivery Platform for Sustained Release of Centella asiatica Extract
Source: Int J Mol Sci. 2025 Dec 17;26(24):12134. doi: 10.3390/ijms262412134 (PMC12733457; doi:10.3390/ijms262412134)
Supplement: Supplementary file 1 [file ijms-26-12134-s001.zip › ijms-4018069-supplementary.pdf]

International Journal of *Molecular Sciences*

MDPI

Nanostructured Chitosan Fibers as a Delivery Platform for Sustained Release of *Centella asiatica* Extract

Table S1. Raw asiaticoside release data

Time [days]	Cumulative release [%]	SD
0.020833333	2.37	1.27
0.041666667	5.26	1.47
0.083333333	7.26	2.47
0.166666667	8.63	2.58
0.2	10.92	2.48
1	29.47	2.18
2	42.26	3.17
4	67.63	3.02
7	93.73	3.92
